# Supplementary material for: Carbon Nanotubes Interconnected NiCo Layered Double Hydroxide Rhombic Dodecahedral Nanocages for Efficient Oxygen Evolution Reaction
Source: Nanomaterials (Basel). 2022 Mar 20;12(6):1015. doi: 10.3390/nano12061015 (PMC8951491; doi:10.3390/nano12061015)
Supplement: Supplementary file 1 [file nanomaterials-12-01015-s001.zip › nanomaterials-1615441-supplementary.pdf]

nanomaterials

MDPI

Article

Carbon Nanotubes Interconnected NiCo Layered Double Hydroxide Rhombic Dodecahedral Nanocages for Efficient Oxygen Evolution Reaction

Meng Li [1,2,†], Yujie Huang [2,†], Jiaqi Lin [2], Meize Li [2], Mengqi Jiang [2], Linfei Ding [3], Dongmei Sun [2], Kai Huang [1,*] and Yawen Tang [2,*]

1 School of Chemistry and Chemical Engineering, Southeast University, Nanjing 211189, China; limeng374@163.com

2 Jiangsu Key Laboratory of New Power Batteries, Jiangsu Collaborative Innovation Centre of Biomedical Functional Materials, School of Chemistry and Materials Science, Nanjing Normal University, Nanjing 210023, China; huangyj0206@163.com (Y.H.); linjq0215@163.com (J.L.); meitselee@163.com (M.L.); jiangmengqi666@163.com (M.J.); sundongmei@njnu.edu.cn (D.S.)

3 Advanced Analysis and Testing Center, Nanjing Forestry University, Nanjing 210037, China; jsdinglinfei@163.com

* Correspondence: huangk@seu.edu.cn (K.H.); tangyawen@njnu.edu.cn (Y.T.)

† These authors contributed equally to this work.

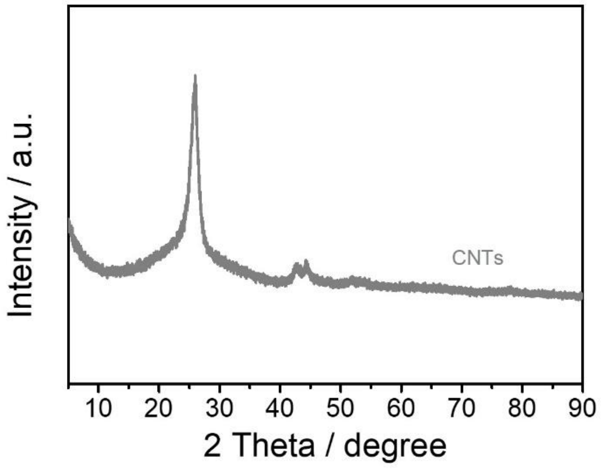

Figure S1. XRD pattern of CNTs.

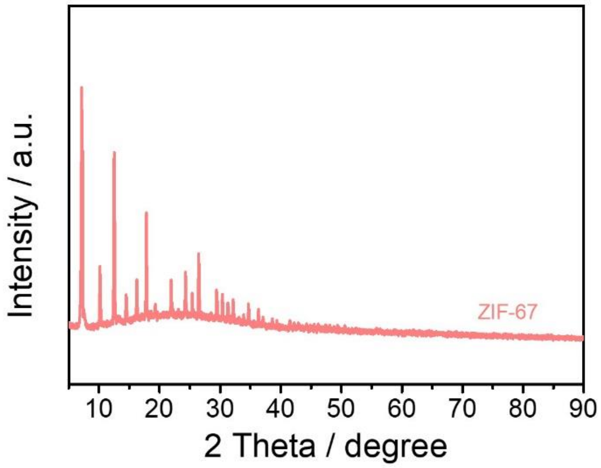

Figure S2. XRD pattern of ZIF-67.

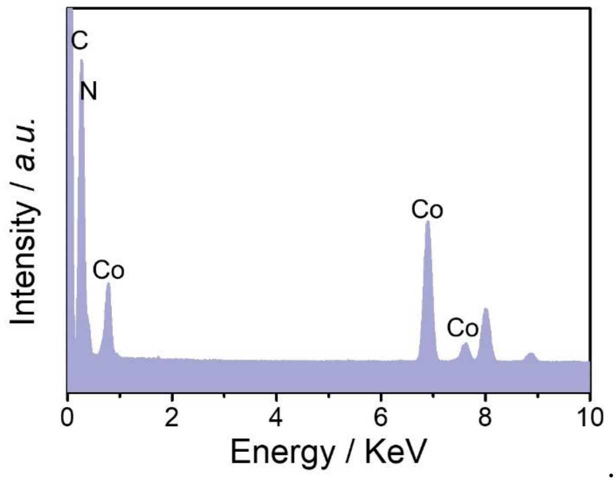

Figure S3. EDS spectrum of ZIF-67@CNTs.

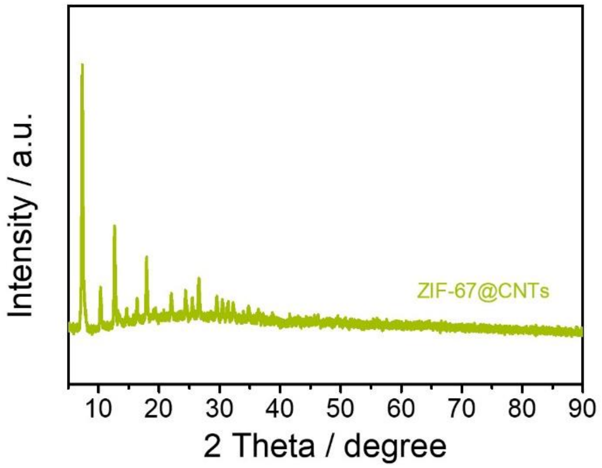

Figure S4. XRD pattern of ZIF-67@CNTs.

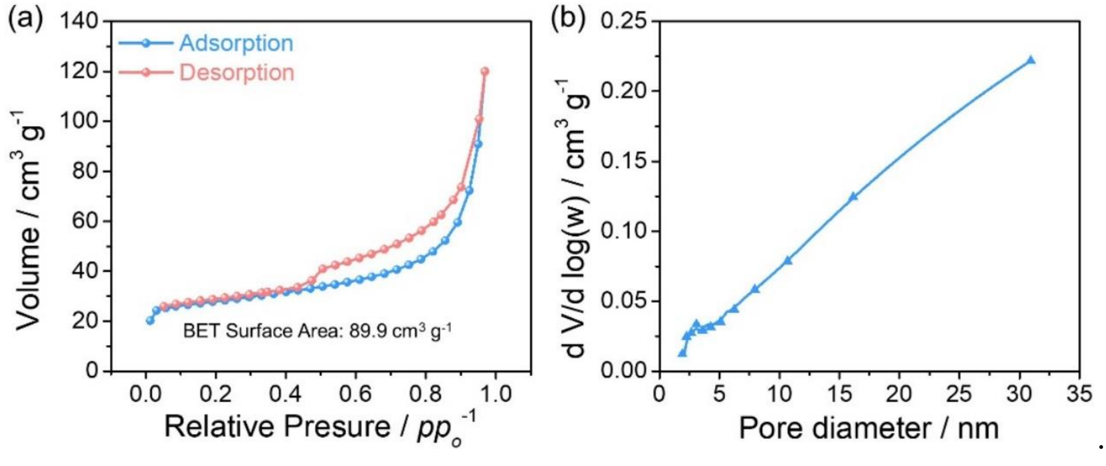

Figure S5. (**a**) N_2 adsorption–desorption isotherms and (**b**) corresponding pore size distribution curve of NiCo-LDH RDC@CNTs.

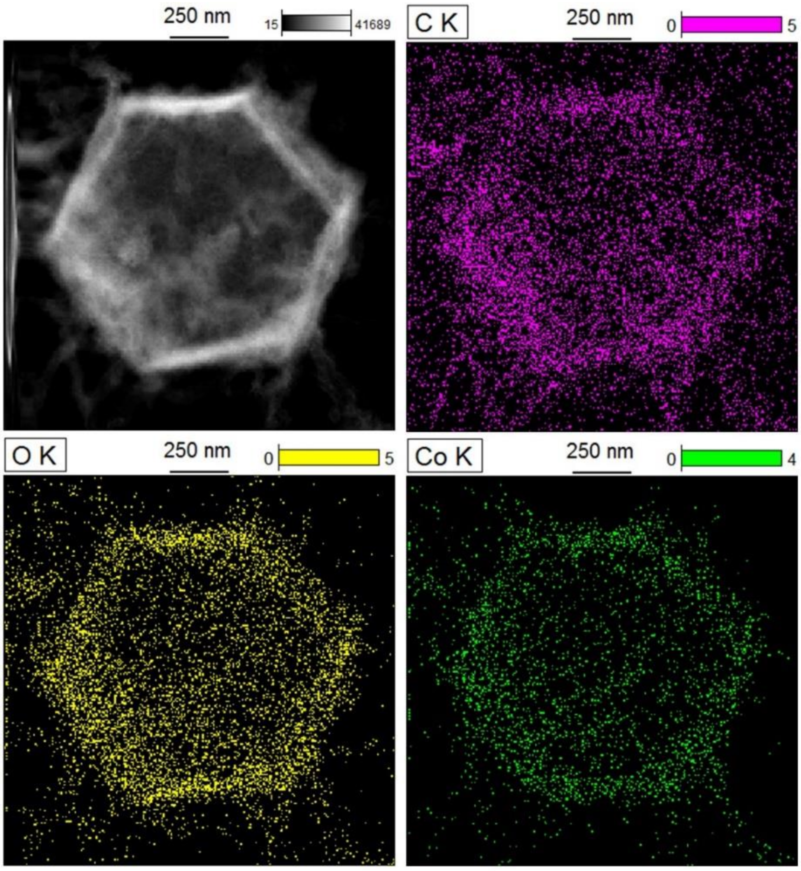

Figure S6. HADDF-STEM image and corresponding EDS elements mapping profiles of NiCo-LDH RDC@CNTs.

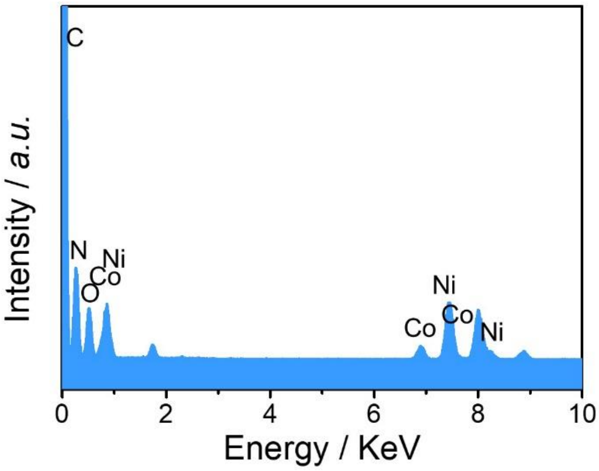

Figure S7. EDS spectrum of NiCo-LDH RDC@CNTs.

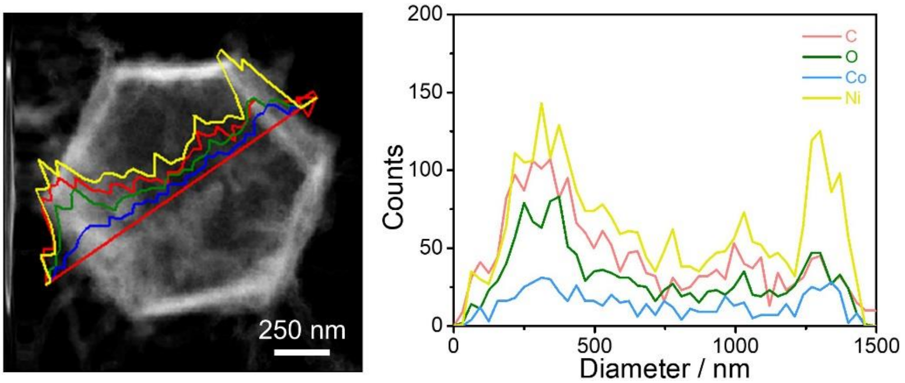

Figure S8. HADDF-STEM image and corresponding EDS linescan profiles of NiCo-LDH RDC@CNTs.

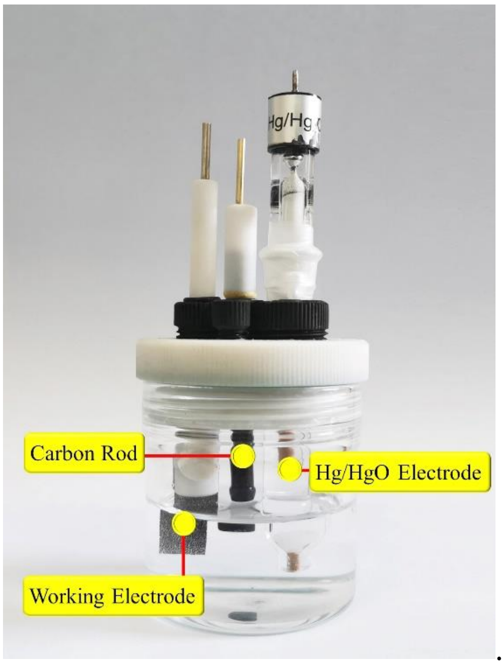

Figure S9. Digital image of OER electrolyzer.

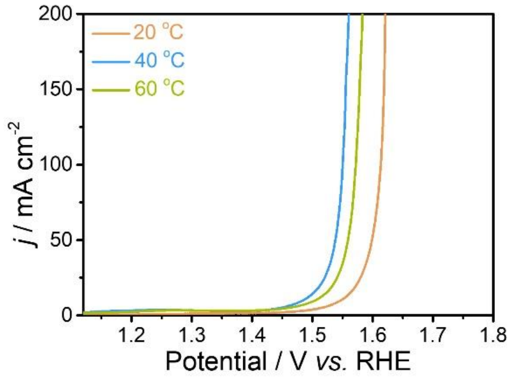

Figure S10. The OER polarization curves of NiCo-LDH RDC@CNTs samples with different reaction temperature (20 °C, 40 °C and 60 °C).

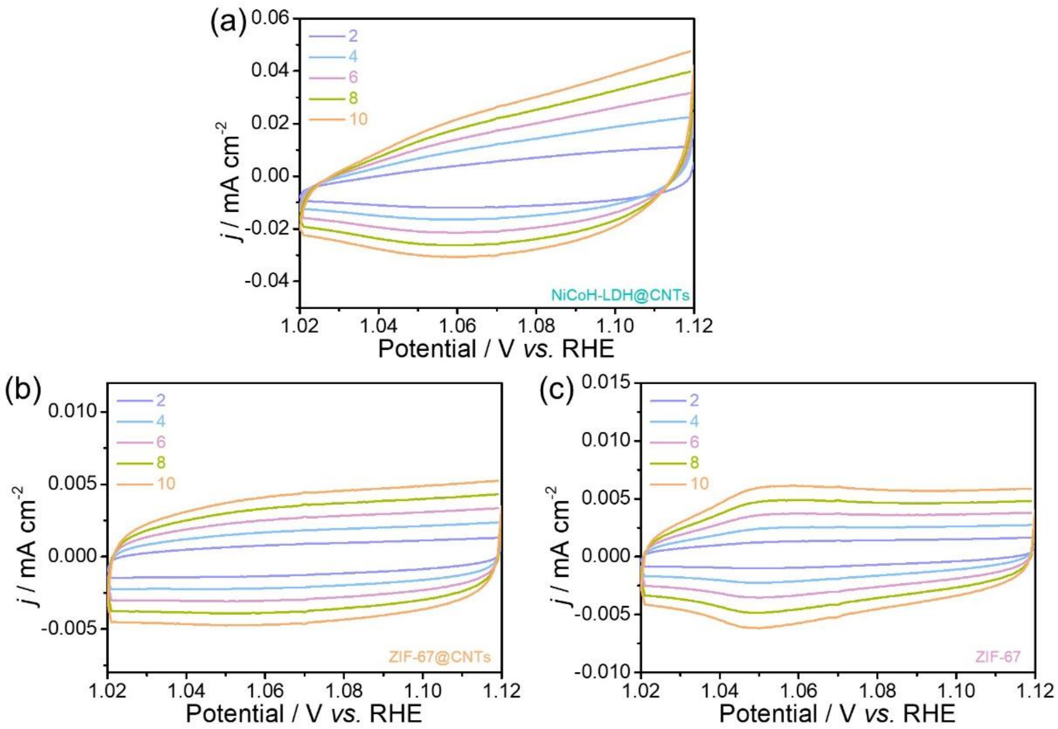

Figure S11. CVs at different sweeping rates from 2 mV s^{-1} to 10 mV s^{-1} of (a) NiCo-LDH RDC@CNTs, (b) ZIF-67@CNTs, (c) ZIF-67.

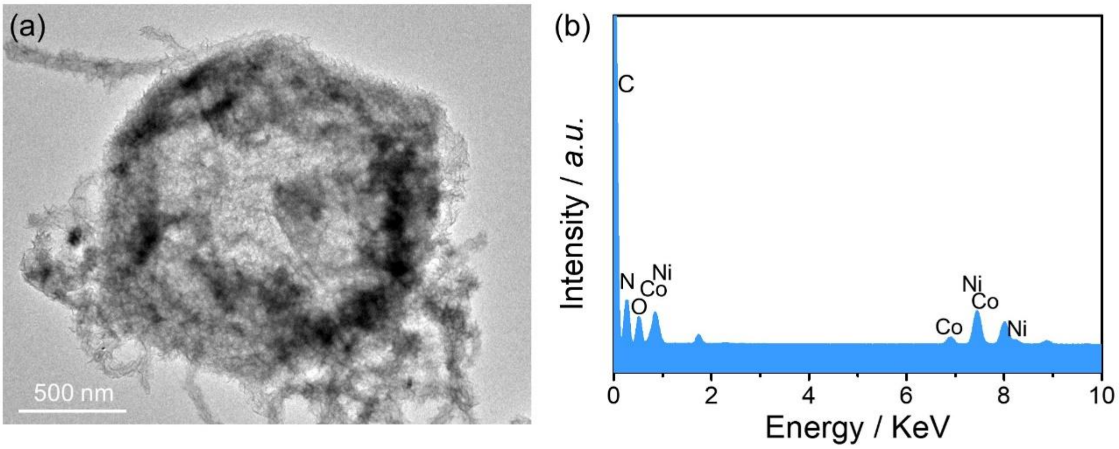

Figure S12. (a) TEM image and (b) EDS profile of recovered NiCo-LDH RDC@CNTs after stability test.

Table S1. Comparison of OER activity of NiCo-LDH RDC@CNTs with other catalysts reported.

**Catalysts**	**η_j / mV at 10 mA cm^{-2}**	**Tafel slope / mV dec^{-1}**	**Reference**
NiCo-LDH RDC@CNTs	255	78.23	This Work
M-Co_3O_4/NPC	302	83	Nano-Micro Lett. (2018) 10:15
GNiPy350N	320	136	J. Mater. Chem. A, 2021, 9, 11255 – 11267
$CuCo_2S_4$	310	86	ACS Catal. 2017, 7, 5871.
graphene@$NiCo_2S_4$	264	80.3	J. Mater. Chem. A 2018, 6, 8109
A-$CoS_{4.6}O_{0.6}$	290	67	Angew. Chem. Int. Ed. 2017, 56, 4858- 4861
Co_3S_4	360	90	ACS Nano. 2014, 8, 10909-10919
FeOOH@$Ni(OH)_2$	310	70	Electrochim. Acta 2019, 301, 258-266
CeO_x/CoO_x	313	66	ACS Catal. 2018, 8, 5, 4257 – 4265
NiCoP/C	330	96	Angew. Chem. Int. Ed. 2017, 56, 3897-3900
Co_9S_8/NSC-900	410	99	NPG Asia Mater. (2018) 10: 670-684
CoZn-NC-700	390	69	Adv. Funct. Mater. 2017, 27, 1700795
